# Pancreatic Cancer Cells Enhance the Ability of Collagen Internalization during Epithelial–Mesenchymal Transition

**DOI:** 10.1371/journal.pone.0040434

**Published:** 2012-07-05

**Authors:** Naoki Ikenaga, Kenoki Ohuchida, Kazuhiro Mizumoto, Shin Akagawa, Kenji Fujiwara, Daiki Eguchi, Shingo Kozono, Takao Ohtsuka, Shunichi Takahata, Masao Tanaka

**Affiliations:** 1 Department of Surgery and Oncology, Graduate School of Medical Sciences, Kyushu University, Fukuoka, Japan; 2 Research Fellow of the Japan Society for the Promotion of Science, Tokyo, Japan; 3 Department of Advanced Medical Initiatives, Graduate School of Medical Sciences, Kyushu University, Fukuoka, Japan; 4 Kyushu University Hospital Cancer Center, Fukuoka, Japan; Wayne State University School of Medicine, United States of America

## Abstract

**Background:**

Extracellular matrix (ECM) remodeling is predominantly mediated by fibroblasts using intracellular and extracellular pathways. Although it is well known that extracellular degradation of the ECM by proteases derived from cancer cells facilitates cellular invasion, the intracellular degradation of ECM components by cancer cells has not been clarified. The aim of this study was to characterize collagen internalization, which is the initial step of the intracellular degradation pathway in pancreatic cancer cells, in light of epithelial–mesenchymal transition (EMT).

**Methodology/Principal Findings:**

We analyzed the function of collagen internalization in two pancreatic cancer cell lines, SUIT-2 and KP-2, and pancreatic stellate cells (PSCs) using Oregon Green 488-gelatin. PSCs had a strong ability for collagen uptake, and the pancreatic cancer cells also internalized collagen although less efficiently. The collagen internalization abilities of SUIT-2 and KP-2 cells were promoted by EMT induced by human recombinant transforming growth factor β1 (*P*<0.05). Expression of Endo180, a collagen uptake receptor, was high in mesenchymal pancreatic cancer cell lines, as determined by EMT marker expression (*P*<0.01). Quantitative RT-PCR and western blot analyses showed that Endo180 expression was also increased by EMT induction in SUIT-2 and KP-2 cells. Endo180 knockdown by RNA interference attenuated the collagen uptake (*P*<0.01) and invasive abilities (*P*<0.05) of SUIT-2 and KP-2 cells.

**Conclusions/Significance:**

Pancreatic cancer cells are capable of collagen internalization, which is enhanced by EMT. This ECM clearance system may be a novel mechanism for cellular invasion and a potential therapeutic target in pancreatic cancer.

## Introduction

Pancreatic cancer is one of the most lethal cancers, and its 5-year survival rate is only 5% [1]. It is characterized by excessive desmoplasia with abundant extracellular matrix (ECM), which plays a crucial role in its aggressive behavior [2], [3]. The ECM within pancreatic tumors is mainly produced by the activated phenotype of pancreatic stellate cells (PSCs), and ECM remodeling by PSCs and pancreatic cancer cells is one of the crucial steps during cancer progression [2], [4], [5]. To date, a number of extracellular proteases derived from cancer cells have been identified as contributors to cancer invasion and progression, including matrix metalloproteinases (MMPs), a disintegrin and metalloproteinases (ADAMs) and urokinase plasminogen activator [6].

Collagens are the most abundant ECM components in the body and undergo continuous synthesis and degradation under physiological and pathological conditions. Stromal fibroblasts, which are the primary cells for maintaining ECM homeostasis, degrade collagen fibrils intracellularly in addition to the extracellular degradation mediated by multiple proteases [7], [8]. The initial step of collagen uptake is binding of collagen to membrane receptors, such as α_2_β_1_-integrin [7], [9] and Endo180 [10], followed by subsequent endocytosis, transportation to lysosomes and degradation by lysosomal cysteine cathepsins [11]. Endo180 is a member of the macrophage mannose receptor family subject to constitutive internalization and recycling [12] and is essential for cellular uptake of collagen in fibroblasts [10]. Recently, Endo180 was found to be expressed in not only fibroblasts but also a subset of neoplasms including glioblastomas [13] and basal-like breast tumors [14].

Epithelial–mesenchymal transition (EMT) is a developmental process in which polarized epithelial cells switch to a highly motile mesenchymal phenotype to undergo multiple biochemical changes [15]. In epithelial cancer, EMT induction is a central process in cancer progression, and provides cancer cells with invasive and metastatic abilities. Reduced expression of E-cadherin, one of the epithelial markers, indicates mesenchymal transition of epithelial cancer cells, and is involved in the poor prognosis of pancreatic cancer [16]. EMT-related molecules are also expected to be therapeutic targets.

Based on these lines of evidence that fibroblasts have a strong ability to degrade collagen and that the mesenchymal phenotype is associated with cancer invasion, we hypothesized that pancreatic cancer cells also internalize collagen, similar to fibroblasts, and enhance their invasive ability by undergoing EMT. To date, extracellular degradation regulated by extracellular proteases, including MMPs, derived from pancreatic cancer cells has been well established. However, the intracellular degradation of collagen by pancreatic cancer cells has not been documented. We investigated whether pancreatic cancer cells have the ability to internalize collagen in an *in vitro* study, and assessed the impact of collagen internalization on pancreatic cancer invasion in light of EMT.

## Results

### Not only PSCs but also pancreatic cancer cells have the ability to internalize collagen

**Figure 1:**
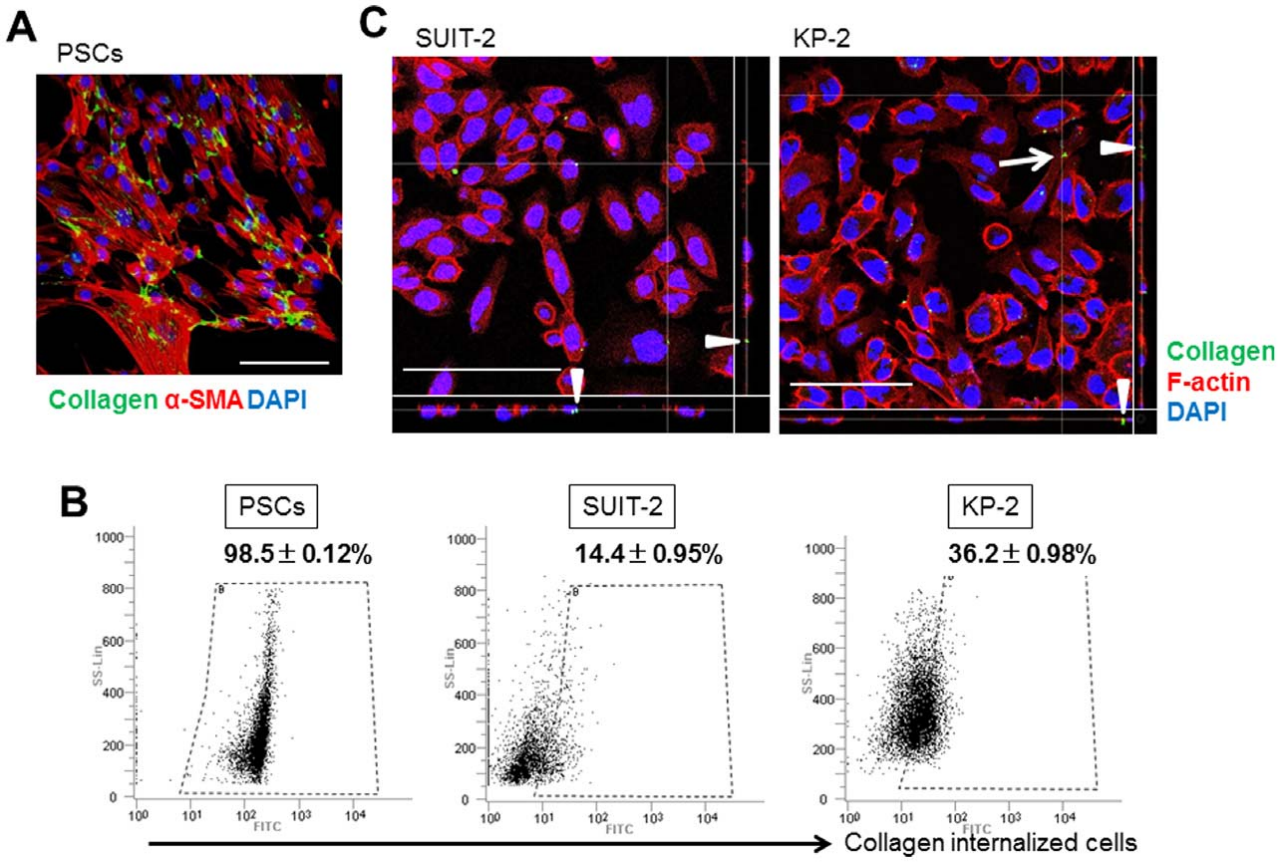
Collagen uptake by PSCs and pancreatic cancer cells. (A) Representative microphotograph of immunofluorescence staining of α-SMA in PSCs. The PSCs have a stellate-like or spindle-shaped morphology, and express α-SMA (red). The PSCs have internalized many collagen molecules (green). Scale bar: 100 µm. Original magnification: ×200. (B) Flow cytometry analyses of PSCs, SUIT-2 cells and KP-2 cells after incubation with OG-gelatin for 2 h. Each sample was analyzed in triplicate, and the percentages of collagen-internalized cells are shown as means ± SD in the representative figures. (C) Confocal microscopy images of collagen in the cytoplasm of SUIT-2 and KP-2 cells. The cells were incubated with OG-gelatin (green), fixed and stained with Alexa Fluor 647-conjugated phalloidin (red) to visualize the cell outlines. Orthogonal sections in the XY, XZ and YZ planes are shown. The Y and Z axes illustrate the localization of the internalized collagen more clearly (arrowheads). In particular, the spindle-shaped cancer cells with a mesenchymal morphology contain many collagen molecules (arrows). Scale bars: 100 µm. Original magnification: ×400.

We established *in vitro* PSCs from resected pancreatic cancers to examine whether PSCs have the function of collagen uptake, similar to fibroblasts. The identity of the activated PSCs was confirmed by immunohistochemical staining for vimentin and α-smooth muscle actin (α-SMA) as described previously [17]. Oregon Green 488-gelatin (OG-gelatin), a denatured form of collagen, was visualized in the cytoplasm of PSCs after incubation for 2 h (Figure 1A), and flow cytometry analyses demonstrated that more than 95% of PSCs had internalized collagen (Figure 1B). The pancreatic cancer cell lines SUIT-2 and KP-2 were also able to internalize native collagen I, although less efficiently (Figure 1B). Confocal microscopy analyses confirmed the intracellular localization of OG-gelatin in pancreatic cancer cells (Figure 1C). In particular, spindle-shaped cancer cells with a mesenchymal morphology seemed to internalize collagen strongly. Cells incubated with fluorescein-conjugated bovine serum albumin (fluorescein-BSA) as a control contained no signals in both fluorescence microphotography and flow cytometry (data not shown).

### EMT in pancreatic cancer cells enhances their collagen internalization

**Figure 2:**
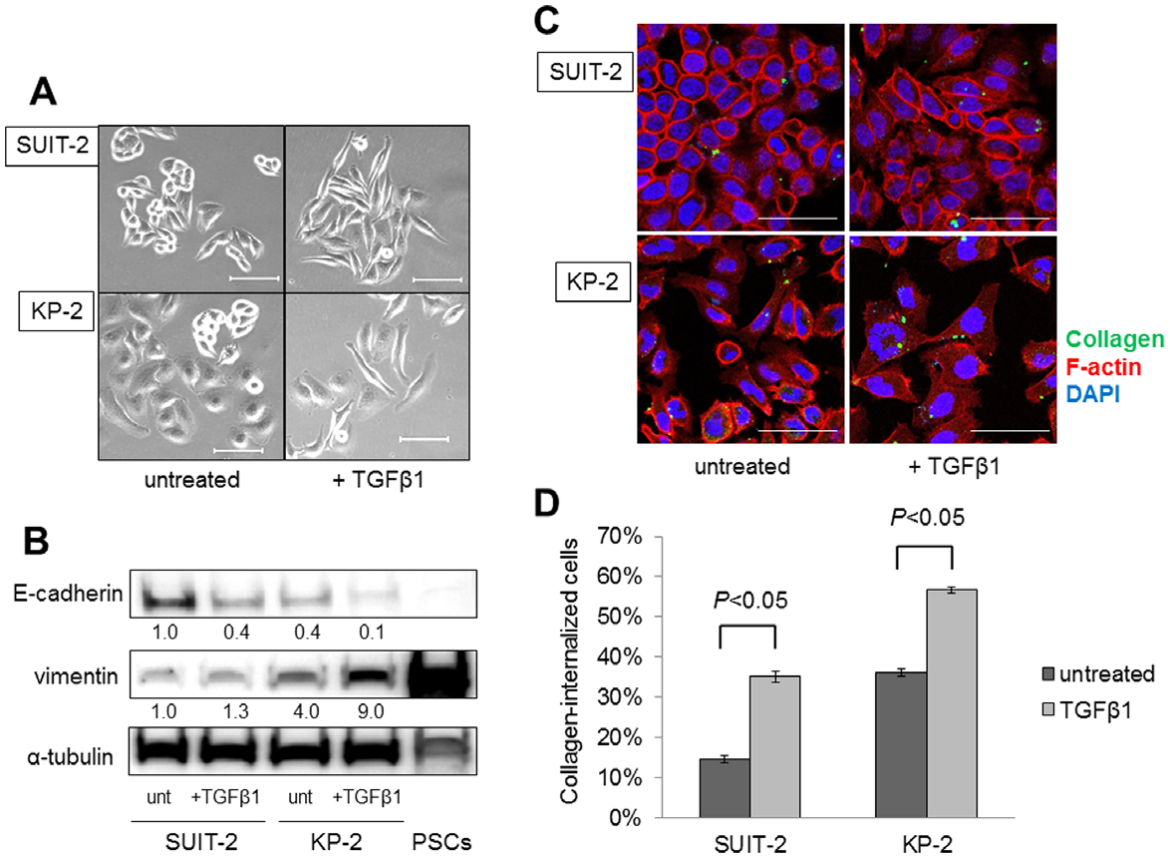
Treatment with TGF-β1 induces EMT in SUIT-2 and KP-2 cells and enhances their collagen internalization. (A) SUIT-2 and KP-2 cells are transformed to a spindle-shaped morphology following treatment with TGF-β1 for 72 h. Scale bars: 100 µm. Original magnification: ×200. (B) Western blotting analysis showing that E-cadherin expression is reduced and vimentin expression is increased in SUIT-2 and KP-2 cells by TGF-β1 treatment. The fold differences in the representative immunoblots quantified by densitometry are shown below the respective lanes. unt: untreated. (C) SUIT-2 and KP-2 cells were cultured with or without TGF-β1 for 72 h, followed by incubation with OG-gelatin (green) for 2 h. The cells were fixed and stained with Alexa Fluor 647-conjugated phalloidin (red) to visualize the cell outlines. Fluorescence microscopy reveals that the collagen internalized by SUIT-2 and KP-2 cells is increased after treatment with TGF-β1. Scale bars: 100 µm. Original magnification: ×400. (D) Quantitative data for collagen internalization by SUIT-2 and KP-2 cells determined by flow cytometry reveals significant differences in the collagen uptake ability between untreated and TGF-β1-treated cancer cells. Each cell line was analyzed in triplicate and the percentages of collagen-internalized cells are expressed as means ± SD. Comparisons between untreated and TGF-β1-treated cells were carried out using Student's *t*-test. All experiments were performed more than three times and representative images are shown.

We investigated whether EMT in pancreatic cancer cells is associated with the function of collagen internalization, because PSCs, which have a mesenchymal phenotype, have a strong ability for collagen internalization. For EMT induction in cancer cells, we used transforming growth factor (TGF)-β1, which is a major factor during EMT with many contributory roles [15]. Pancreatic cancer cells treated with TGF-β1 showed a spindle-shaped fibroblastic morphology and cell scattering compared with untreated cancer cells (Figure 2A). Western blotting analyses showed that E-cadherin expression was reduced and vimentin expression was increased in SUIT-2 and KP-2 cells after treatment with TGF-β1 (Figure 2B). These findings indicate that the pancreatic cancer cells altered to a mesenchymal phenotype, meaning that EMT was induced by TGF-β1. The collagen internalization abilities of SUIT-2 and KP-2 cells were promoted by EMT induction with TGF-β1 treatment for 72 h (*P*<0.05; Figure 2C, 2D).

### Endo180 expression is associated with the mesenchymal phenotype of pancreatic cancer cells

**Figure 3:**
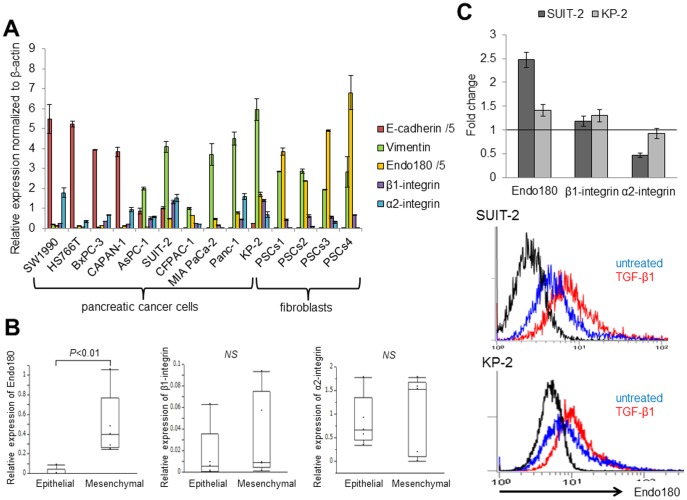
Evaluation of the correlation between EMT induction and expression of collagen receptors in pancreatic cancer cell lines. (A) Relative expression levels of E-cadherin, vimentin, Endo180, β1-integrin and α2-integrin in 10 pancreatic cancer cell lines and four PSC cultures. The expression levels of all genes were normalized by the corresponding expression levels of β-actin. Each sample was analyzed in triplicate, and the data are expressed as means ± SD. One-fifth of the acquired levels for E-cadherin and Endo180 are shown in the graph for ease of reference. (B) The pancreatic cancer cell lines were divided into two groups showing an epithelial phenotype (SW1990, HS766T, BxPC-3, CAPAN-1 and AsPC-1) and a mesenchymal phenotype (SUIT-2, CFPAC-1, MIA PaCa-2, Panc-1 and KP-2) on the basis of the expression ratio of E-cadherin to vimentin. Endo180 is more highly expressed in the cell lines with the mesenchymal phenotype than in those with the epithelial phenotype, while the expressions of β1-integrin and α2-integrin are not correlated with the cell phenotypes. Statistical analyses were performed using the Mann–Whitney *U* -test. (C) SUIT-2 and KP-2 cells were incubated with or without TGF-β1 for 72 h, and the expression levels of Endo180, β1-integrin and α2-integrin were determined by quantitative RT-PCR (upper panel). The expression levels of all genes were normalized by the corresponding expression levels of 18S rRNA. The fold changes were calculated as the mRNA levels in TGF-β1-treated cells divided by the levels in untreated cells. Endo180 mRNA expression in SUIT-2 and KP-2 cells is more highly increased by EMT induction than β1-integrin and α2-integrin mRNA expression. Increased cell surface expression of Endo180 in EMT-induced SUIT-2 and KP-2 cells (red lines) compared with untreated SUIT-2 and KP-2 cells (blue lines), respectively, was confirmed by flow cytometry analyses (*P*<0.01 for each; lower panels). Each sample was analyzed in triplicate, and comparisons between untreated and TGF-β1-treated cells were performed using Student's *t*-test.

Next, we analyzed the expression levels of EMT markers, including E-cadherin and vimentin, the major collagen receptor α2β1-integrin and the collagen uptake receptor Endo180 in the pancreatic cancer cell lines to clarify the relationship between EMT and collagen internalization. The pancreatic cancer cell lines expressed various levels of the EMT markers, α2β1-integrin and Endo180 (Figure 3A). First, we used the ratio of E-cadherin to vimentin to classify the cells as epithelial or mesenchymal phenotype, and then compared the levels of α2-integrin, β1-integrin and Endo180 expression between the cells classified into the two phenotypes. Endo180 was more highly expressed in the mesenchymal pancreatic cancer cell lines than in the epithelial pancreatic cancer cell lines, while the expression of β1-integrin and α2-integrin was not correlated with the cell phenotypes (*P*<0.01; Figure 3B). The expression of Endo180 mRNA in SUIT-2 and KP-2 cells was upregulated by EMT induction with TGF-β1 treatment, together with increases in the cell surface expression of Endo180 from 9.3±0.1% to 19.9±2.4% in SUIT-2 cells and from 19.1±1.0% to 24.8±1.5% in KP-2 cells (*P*<0.01, Figure 3C).

### Knockdown of Endo180 in pancreatic cancer cells attenuates their collagen uptake and invasive abilities

**Figure 4:**
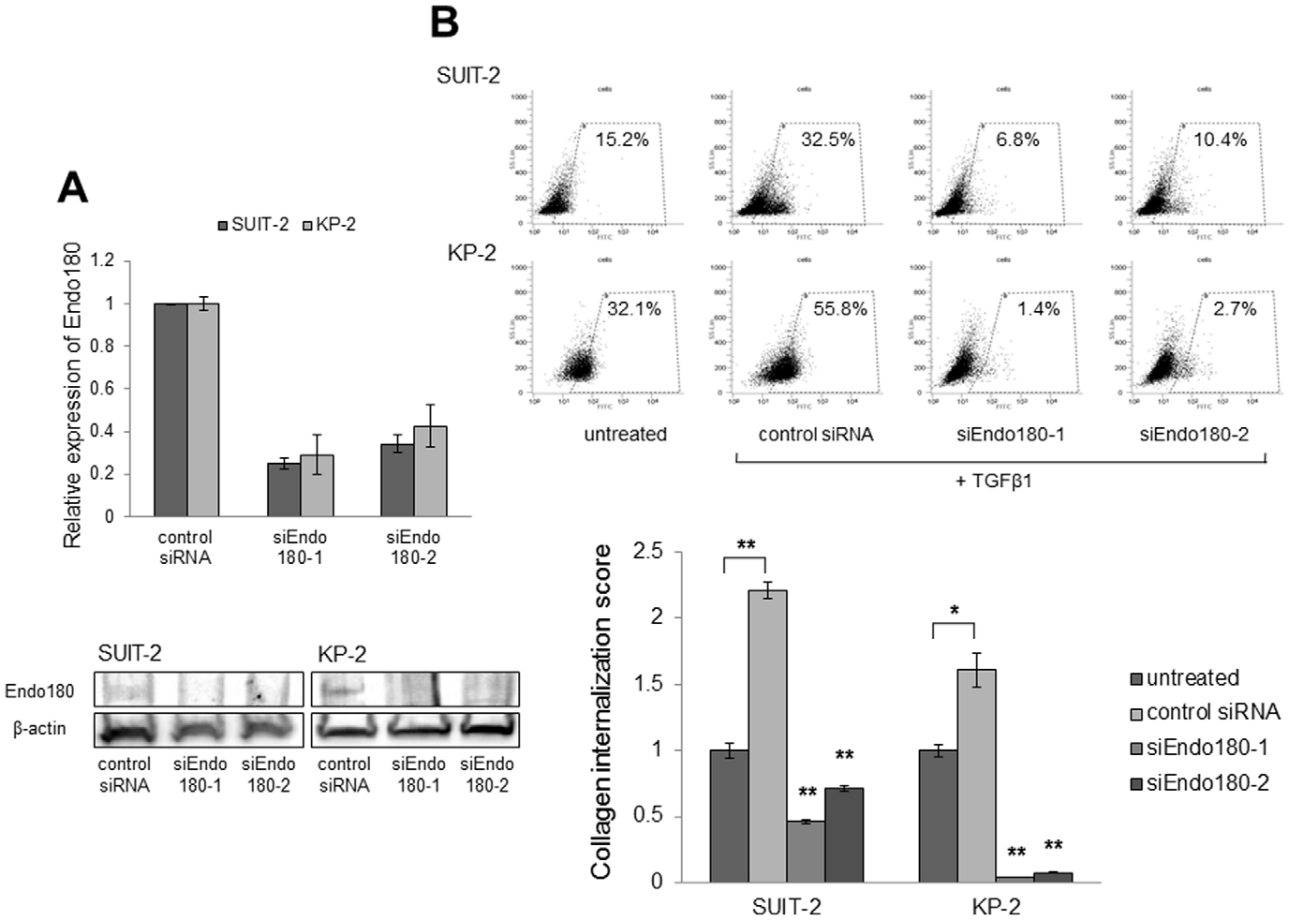
Collagen internalization by pancreatic cancer cells is dependent on Endo180 expression. (A) SUIT-2 and KP-2 cells were transfected with siEndo180-1 or siEndo180-2, and knockdown of Endo180 expression compared with control siRNA-transfected cells was confirmed by quantitative RT-PCR (upper panel) and western blotting analysis (lower panel) for at least 3 days. (B) Following EMT induction with 10 ng/ml TGF-β1, SUIT-2 and KP-2 cells were incubated with OG-gelatin for 2 h. The percentages of OG-gelatin-internalized cells were evaluated by flow cytometry. The dot plots show representative flow cytometry data for untreated SUIT-2 and KP-2 cells and cells transfected with control siRNA, siEndo180-1 or siEndo180-2 (upper panels). The collagen internalization scores represent the relative ratios of collagen-internalized cells among cells transfected with control siRNA, siEndo180-1 or siEndo180-2 to those in untreated cells. The enhanced collagen internalization abilities in SUIT-2 and KP-2 cells after TGF-β treatment are dramatically reduced by knockdown of Endo180 expression (lower panel). Each sample was analyzed in triplicate, and comparisons between TGF-β1-treated cells transfected with control siRNA and other cells were performed using Student's *t*-test. *P<0.01; **P<0.001.

**Figure 5:**
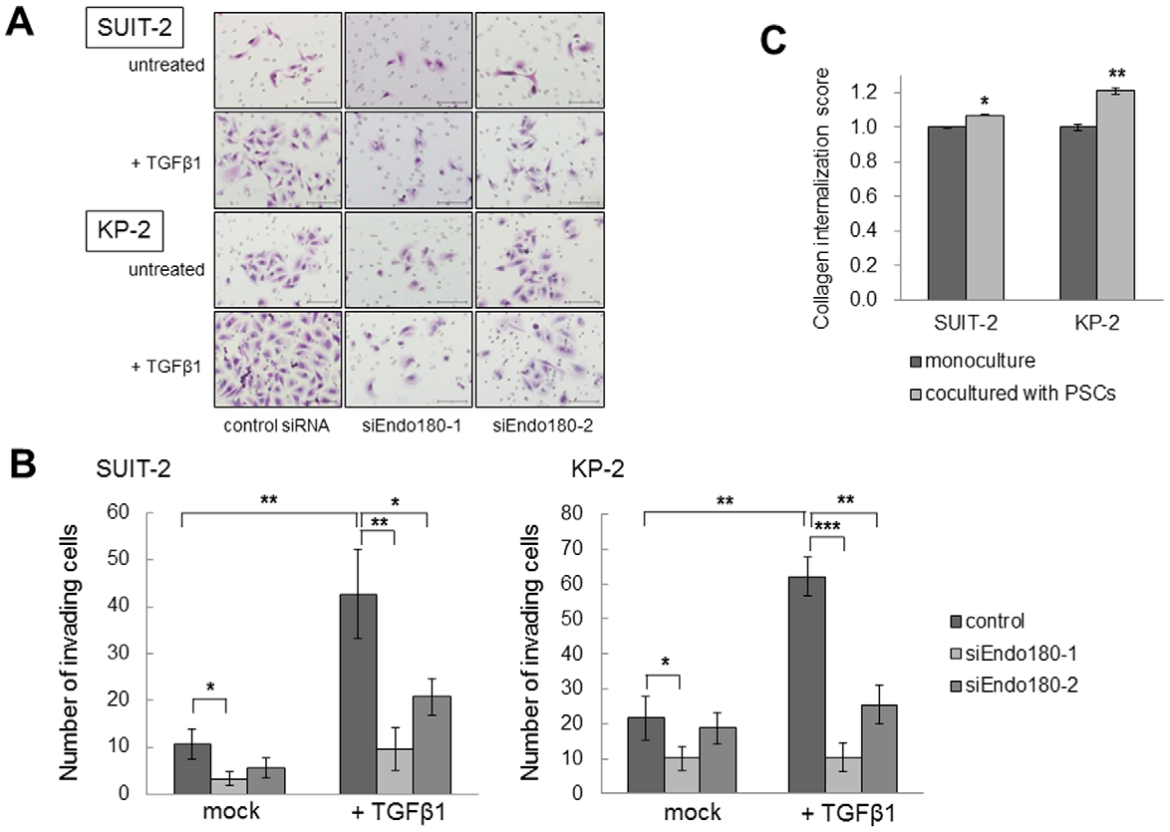
Effects of Endo180 expression by pancreatic cancer cells on their invasive ability. SUIT-2 and KP-2 cells (4×10^4^ cells/well) transected with siRNAs were suspended in 50 µg of 3D type I collagen matrix, and seeded in the upper chambers with or without TGF-β1. After gel formation by incubation at 37°C for 30 min, the upper chambers were placed in the wells of a 24-well culture dish containing 750 µl of 10%FBS/DMEM. The cells that invaded to the lower surface of each upper chamber membrane through the collagen I matrix were fixed, stained with hematoxylin and eosin, and counted. (A) Representative photomicrographs of invading SUIT-2 and KP-2 cells transfected with control siRNA, siEndo180-1 or siEndo180-2. Scale bars, 100 µm. Original magnification, ×200. (B) The invasive abilities of SUIT-2 and KP-2 cells are increased by EMT induction, but attenuated by knockdown of Endo180 expression. Each sample was analyzed in triplicate, and comparisons between two groups were performed using Student's *t*-test. *P<0.05; **P<0.01; ***P<0.001. (C) Collagen internalization scores in SUIT-2 and KP-2 cells after coculture with PSCs for 72 h. The collagen internalization abilities are slightly enhanced in SUIT-2 and KP-2 cells via cancer–stroma interactions. The experiments were performed in triplicate, and comparisons between the data were performed using Student's *t*-test. *P<0.05; **P<0.01.

To investigate whether Endo180 expression is associated with collagen uptake by pancreatic cancer cells, we knocked down Endo180 mRNA in SUIT-2 and KP-2 cells using RNA interference technology. Transient transfection of siEndo180-1 and siEndo180-2 decreased Endo180 expression at both the mRNA and protein levels (Figure 4A). The cell morphologies of SUIT-2 and KP-2 cells did not change after Endo180 knockdown. The collagen internalization abilities of SUIT-2 and KP-2 cells under treatment with TGF-β1 were dramatically attenuated by the suppression of Endo180 expression (*P*<0.01; Figure 4B). In addition, 3D collagen matrix assays showed that the EMT-induced cancer cells displayed a less invasive phenotype when Endo180 expression was suppressed with siRNA. TGF-β1 still enhanced invasion of cancer cells in the presence of control siRNA (Figure 5A, 5B). On the other hand, Endo180 expression was not involved in the cell proliferation and migration of SUIT-2 and KP-2 cells (data not shown).

### PSCs enhance the collagen internalization by pancreatic cancer cells

Finally, we investigated whether PSCs had an impact on the collagen internalization by pancreatic cancer cells, since PSCs are known to promote the progression and EMT of pancreatic cancer via cancer–stroma interactions [18], [19]. The collagen internalization abilities of SUIT-2 and KP-2 cells cocultured with PSCs were slightly enhanced compared with those in the monocultured cancer cells (*P*<0.05 and *P*<0.01, respectively; Figure 5C). These findings suggest that cancer–stroma interactions also promote collagen uptake by pancreatic cancer cells.

## Discussion

Invasion of cancer cells, a critical step in cancer progression, consists of ECM degradation and subsequent migration of cancer cells to newly formed extracellular spaces. MMPs have been regarded as the major proteases capable of degrading various ECM components to assist tumor progression, and are considered to be promising targets for cancer therapy [20], [21]. However, most clinical trials of MMP inhibitors have yielded disappointing results with limited success [22], [23]. In this study, we have shown that not only extracellular degradation of the ECM but also intracellular uptake of ECM components may be involved in cellular invasion. A proteolysis system involving degradation of internalized collagens by lysosomal cysteine cathepsins has also been observed in several malignancies and was reported to be linked to tumor invasion and metastasis [24], [25], [26]. Quintanilla-Dieck et al. [27] showed that cathepsin K in melanoma regulates invasion by mediating intracellular degradation of ECM components. Immunohistochemical analyses of pancreatic cancer tissues revealed that expressions of cathepsin B and cathepsin L are indicators of a poor prognosis [28]. These lines of evidence may suggest that for cellular invasion, it is necessary to create a space to migrate into by internalizing and degrading ECM components intracellularly in addition to their pericellular degradation. The clearance system of ECM components exhibited by cancer cells is potentially a novel mechanism for invasion in pancreatic cancer.

EMT is considered to be a crucial step in cancer progression, although its significance and relevance have remained a matter of debate [15]. Loss of cell–cell adhesion, changes in cell shape and increased motility lead to invasion of cancer cells through the basement membrane into the stromal tissue [15], [29]. EMT-induced cells are typically seen at the invasive front of primary tumors, and develop into subsequent steps of deep migration into the ECM, intravasation, transportation through the circulation and formation of micrometastases [30]. In colorectal cancer, small aggregates of tumor cells extending from the tumor mass into the adjacent stroma have been shown as morphological evidence of EMT at the invasive fronts of human cancer [31]. Rhim et al. [32] showed that tagged malignant epithelial cells invaded into the stroma via EMT and intermingled with stromal cells using a genetic mouse model with spontaneous pancreatic cancer. We found that EMT-induced pancreatic cancer cells might promote their invasive ability by not only increasing motility, but also enhancing collagen internalization. Collagen internalization may be one of the contributing factors to cancer progression provided by EMT, and these cell populations capable of collagen uptake may be leading cells for invasion. In fibroblasts, α_2_β_1_-integrin is considered to be important for cellular interactions with collagen and has been proposed to play a role in the internalization process [33]. However, in our study, α_2_β_1_-integrin expression was not involved in the cell phenotypes of pancreatic cancer cell lines and was not increased in EMT-induced SUIT-2 and KP-2 cells. According to experiments using an anti-β_1_-integrin antibody and Endo180-deficient fibroblasts [8], the Endo180-mediated adhesion of fibroblasts to collagen matrices is a β_1_-integrin-independent process. Taken together, cellular invasion enhanced by EMT could be attributed to Endo180 alone and not to α_2_β_1_-integrin.

The desmoplasia in pancreatic cancer manifests as bands of fibrous stroma surrounding cancer cells, resulting in an increase in fibrillar collagens. This fibrous stroma is predominantly composed of collagen type I, which was reported to promote proliferation, migration and chemoresistance in pancreatic cancer cells [2], [34]. On the other hand, tumor-prone mice with Endo180-deficient fibroblasts showed increased accumulation of collagen within the tumors [35], and mice implanted with breast cancer cells expressing an internalization-defective Endo180 mutant had increased intratumoral fibrosis [14]. These findings were correlated with a decrease in collagen internalization and reduced collagen turnover, eventually resulting in restricted tumor growth. In our study, PSCs had a strong ability for collagen internalization, and Endo180-sufficient pancreatic cancer cells showed stronger invasion than Endo180-deficient cells. Therefore, ECM turnover mediated by Endo180 may be crucial for tumor expansion and cancer progression.

In conclusion, not only mesenchymal PSCs but also pancreatic cancer cells have the ability to internalize collagen. Collagen internalization by cancer cells was enhanced in association with EMT. Knockdown of the collagen uptake receptor Endo180 abrogated collagen internalization by cancer cells and reduced their invasive abilities. This formerly unappreciated invasive mechanism of pancreatic cancer cells needs to be further clarified to identify novel therapeutic targets for pancreatic cancer.

## Materials and Methods

### Cells and culture conditions

The following 10 pancreatic cancer cell lines were used: AsPC-1, KP-2, Panc-1, SUIT-2, BxPC-3 (Dr. H. Iguchi, National Shikoku Cancer Center, Matsuyama, Japan), MIA PaCa-2 (Japanese Cancer Resource Bank), CAPAN-1, CFPAC-1, HS766T and SW1990 (American Type Culture Collection). Human PSCs were isolated from fresh pancreatic cancer surgical specimens using the outgrowth method as described previously [36], [37]. Primary cultures of human PSCs derived from four patients with invasive pancreatic cancers were established in our laboratory with written informed consent. The study was approved by the Ethics Committee of Kyushu University and conducted according to the Ethical Guidelines for Human Genome/Gene Research enacted by the Japanese Government and the Helsinki Declaration. The identity of the PSCs was confirmed by immunofluorescence staining for α-SMA (positive rate: almost 100%) and vimentin (positive rate: almost 100%) and the morphology (stellate-like or spindle-shaped cells) [17]. All cells were maintained in Dulbecco's modified Eagle's medium (DMEM) (Sigma) supplemented with 10% fetal bovine serum (FBS), streptomycin (100 µg/ml) and penicillin (100 µg/ml) at 37°C in a humidified atmosphere containing 10% CO_2_.

### Collagen internalization assay

Cells were seeded in 90-mm dishes at a density of 1×10^5^ cells/dish and incubated in 1%FBS/DMEM with or without 10 ng/ml recombinant human TGF-β1 (R&D Systems) for 72 h. Next, the cells were incubated with 20 µg/ml OG-gelatin in 1%FBS/DMEM for 2 h at 37°C. For quenching of extracellular non-internalized OG-gelatin, the cells were incubated with 0.4% trypan blue (Sigma) in saline solution for 5 min. After three washes with cold PBS, the cells were detached by trypsinization, and subjected to flow cytometry to determine the proportion of cells that had incorporated collagen. As a control, 20 µg/ml fluorescein-BSA (Molecular Probes) was evaluated using the same protocol. In the collagen internalization analyses for cancer cells transfected with siRNAs, SUIT-2 and KP-2 cells were transfected with control siRNA, siEndo180-1 or siEndo180-2, seeded in 90-mm dishes at 4×10^5^ cells/dish and treated with 10 ng/ml TGF-β1 for 48 h to induce EMT. The cells were then incubated with 20 µg/ml OG-gelatin or fluorescein-BSA in 1%FBS/DMEM for 2 h at 37°C, and non-internalized OG-gelatin or fluorescein-BSA was quenched by incubation with 0.4% trypan blue. After washing with cold PBS, the cells were harvested and analyzed by flow cytometry. The collagen internalization score was calculated as the relative ratio of collagen-internalized cells among cells transfected with control siRNA, siEndo180-1 or siEndo180-2 to those in untreated cells.

### Laser-scanning confocal microscopy for immunofluorescence staining

Cells were plated on 35-mm Glass Bottom Dishes (Matsunami) at a density of 2×10^4^ cells/dish and incubated in 1%FBS/DMEM with or without 10 ng/ml TGF-β1 for 48 h. Next, the cells were incubated with 20 µg/ml OG-gelatin or fluorescein-BSA in 1%FBS/DMEM for 2 h at 37°C, followed by fixation with 4% paraformaldehyde for 10 min at room temperature after quenching of extracellular fluorescein with 0.4% trypan blue. SUIT-2 and KP-2 cells were then incubated with Alexa Fluor 647-conjugated phalloidin (Molecular Probes) to stain F-actin for visualization of the cell outlines according to the manufacturer's instructions, while PSCs were incubated with a rabbit anti-α-SMA antibody (Epitomics; 1∶100 dilution) for 2 h, followed by incubation with Alexa Fluor 647-conjugated anti-rabbit IgG (Molecular Probes) for 1 h. Nuclear DNA was counterstained with 0.05 µg/ml 4′,6-diamidino-2-phenylindole. A laser-scanning confocal fluorescence microscope (A1R; Nikon) was used for immunofluorescence microphotography. Images were managed using NIS-Elements software (Nikon).

### Isolation of RNA

Total RNA was extracted from cultured cells using a High Pure RNA Isolation Kit (Roche Diagnostics) with DNase I (Roche Diagnostics) treatment. The extracted RNA was quantified by the absorbance at 260 nm, and its purity was evaluated from the 260/280 ratio of absorbance with a NanoDrop ND-1000 spectrophotometer (NanoDrop Technologies).

### Quantitative reverse-transcription-polymerase chain reaction (RT-PCR)

Quantitative RT-PCR was performed using a QuantiTect SYBR Green Reverse-Transcription PCR Kit (Qiagen) and a Chromo4 Real-Time PCR Detection System (Bio-Rad Laboratories). We designed specific primers for E-cadherin and vimentin using Primer 3 software and performed BLAST searches to ensure the primer specificities. Primers for Endo180, β1-integrin, α2-integrin, β-actin and 18S rRNA were purchased from Takara Bio Inc. The sequences of the primers used in the present study are shown in Table 1. Each reaction mixture was first incubated at 50°C for 30 min to allow reverse transcription, in which first-strand cDNA was synthesized by priming the total RNA with a gene-specific primer. PCR was initiated by incubation at 95°C for 15 min to activate the polymerase, followed by 40 cycles of 94°C for 15 s, 55°C for 30 s and 72°C for 30 s (for E-cadherin and vimentin) or 95°C for 5 s, 60°C for 20 s and 72°C for 30 s (for Endo180, β1-integrin, α2-integrin, β-actin and 18S rRNA). The expression levels of the genes were calculated based on standard curves constructed with total RNA from SUIT-2 cells. The expression levels were normalized by the β-actin or 18S rRNA expression and expressed as the ratio of expression of the target gene to that of β-actin or 18S rRNA. All samples were run in triplicate. No detectable PCR products were amplified without prior reverse transcription. The accuracy and integrity of the PCR products were confirmed with an Agilent 2100 Bioanalyzer (Agilent Technologies Inc.).

**Table 1:** Primers used for qRT-PCR.

Primer	Forward Sequence 5′-3′	Reverse Sequence 5′-3′
E-cadherin	tcagcgtgtgtgactgtgaa	aggctgtgccttcctacaga
Vimentin	tgcccttaaaggaaccaatg	gcttcaacggcaaagttctc
Endo180	cgcttgcaccaacatcacc	gggagaagctgctctgctc
β1-integrin	tcagcagtaatgcaaggccaataa	acgaggtcatggttcatgttgtg
α2-integrin	agacatcatcatacagaaggcagga	tgggtatgcctgaatgtacacaaa
β-actin	tggcacccagcacaatgaa	ctaagtcatagtccgcctagaagca
18S rRNA	actcaacacgggaaacctca	aaccagacaaatcgctccac

### Flow cytometry

Cultured cells were harvested by exposure to trypsin/EDTA for 5 min at 37°C, and washed in 10%FBS/DMEM. The obtained single cells were suspended in ice-cold 1% FBS/PBS solution and analyzed using a flow cytometer (EC800; SONY) equipped with a laser that provided an excitation wavelength of 488 nm. Eclipse Analysis software (SONY) was used to quantify the fluorescent signals and set the logical electronic-gating parameters.

### Invasion assay in a 3D collagen I matrix and migration assay

The invasiveness of pancreatic cancer cells was assessed based on the number of cells invading through transwell chambers (BD Biosciences) under 3D culture conditions in a collagen I matrix. The collagen I matrix gel was prepared by dilution of Rat Tail Collagen Type I (BD Biosciences) in 10%FBS/DMEM (1∶10) including 0.023 N NaOH (final collagen concentration: 0.38 µg/µl). Cancer cells (4×10^4^ cells/well) were suspended in 50 µl of collagen I matrix solution and seeded in the upper chambers with 8-µm pores. After allowing gelation by incubation at 37°C for 30 min, the upper chambers were placed in the wells of a 24-well culture dish containing 750 µl of 10%FBS/DMEM. For EMT induction, 10 ng/ml TGF-β1 was added to both the collagen I matrix solution and the medium in the lower chambers. After incubation for 48 h, the numbers of cancer cells that had invaded to the lower surface of each upper chamber membrane were counted in five random fields at 200× magnification under a light microscope (TE2000; Nikon). The results were expressed as the mean number of invading cells. Each experiment was carried out in triplicate wells, and independent experiments were repeated twice.

The migration ability of pancreatic cancer cells was assessed based on the number of cells migrating through transwell chambers as described previously [38]. Briefly, cancer cells (4×10^4^ cells/well) were seeded in the upper chambers with 8-µm pores and incubated in the wells of a 24-well dish containing 10%FBS/DMEM for 24 h. The numbers of cancer cells that had migrated to the lower surface of each upper chamber membrane were counted. Each experiment was carried out in triplicate wells, and independent experiments were repeated more than twice.

### Propidium iodide (PI) assay

Cell proliferation was evaluated by measuring the fluorescence intensity of PI as described previously [39]. Briefly, cells were seeded in 24-well plates (Becton Dickinson) at a density of 1×10^4^ cells/well in triplicate. After incubation for various times (24, 72 or 120 h), PI (30 µM) and digitonin (600 µM) were added to each well to label all nuclei with PI. The fluorescence intensity of PI, corresponding to the total cell number, was measured using an infinite F200 (TECAN).

### Silencing of Endo180 by siRNA

SUIT-2 and KP-2 cells at 90% confluence were transfected with siEndo180-1 (sense, 5′-gcaccagcaacauauccaatt-3′; antisense, 5′-uuggauauguugcuggugcgg-3′) or siEndo180-2 (sense, 5′-cccgaaaccggcuauucaatt-3′; antisense, 5′-uugaauagccgguuucgggag-3′) siRNA (Qiagen) by electroporation using a Nucleofector System (Amaxa Biosystems) according to the manufacturer's recommendations. To verify the specificity of the knockdown effects, we used a control siRNA (Qiagen). The transfected cells were used in subsequent experiments at 24–96 h after transfection.

### Western blotting analysis

SUIT-2 and KP-2 cells (1×10^5^) were incubated with or without 10 ng/ml TGF-β1 in 10%FBS/DMEM in 90-mm dishes for 72 h. SUIT-2 and KP-2 cells transfected with siEndo180-1, siEndo180-2 or control siRNA were incubated in 10%FBS/DMEM for 24 h, followed by incubation in fresh medium containing 10 ng/ml TGF-β1 for 48 h. All cells were lysed with PRO-PREP (iNtRON Biotechnology), and aliquots of the lysates (15 µg protein) were fractionated in 4–15% Mini-PROTEAN TGX Gels (Bio-Rad Laboratories) after boiling for 10 min. The separated proteins were transferred to 0.2-µm polyvinylidene difluoride membranes using a Trans-Blot Turbo™ (Bio-Rad Laboratories). The membranes were incubated with a rabbit anti-E-cadherin antibody (Cell Signaling; 1∶1000 dilution), mouse anti-vimentin antibody (Abcam; 1∶2000 dilution), rabbit anti-Endo180 antibody (Abcam; 1∶500 dilution), mouse anti-α-tubulin antibody (Millipore) or rabbit anti-β-actin antibody (Abcam) overnight at 4°C, and then probed with horseradish peroxidase-conjugated anti-mouse or anti-rabbit IgG (Santa Cruz Biotechnology) for 1 h. The bound antibodies were detected by enhanced chemiluminescence using a ChemiDoc XRS (Bio-Rad Laboratories). Quantity One software (Bio-Rad Laboratories) was used to quantify the chemiluminescent signals.

### In vitro coculture system

SUIT-2 or KP-2 cells (2×10^4^) were seeded into the lower wells of a transwell cell culture system (6-well type; Becton Dickinson). After incubation for 24 h, PSCs (4×10^4^) were seeded into the upper chambers with 3-µm pores, followed by culture in 10%FBS/DMEM for 72 h. After removal of the upper chambers containing the PSCs, the cancer cells were incubated with OG-gelatin (20 µg/ml) in 1%FBS/DMEM for 2 h at 37°C. After quenching of non-internalized OG-gelatin, the cells were washed in cold PBS, harvested and analyzed by flow cytometry. The collagen internalization score was calculated as the relative ratio of collagen-internalized cells cocultured with PSCs to that in monocultured cells.

### Statistical analysis

For *in vitro* experiments, the values are expressed as the mean ± SD. Comparisons between two groups were carried out using Student's *t*-test. All experiments were performed at least twice. Statistical significance was defined as a value of *P*<0.05. All statistical analyses were performed with JMP 8 software (SAS Institute).
